# Expansion of the HSV-2–specific T cell repertoire in skin after immunotherapeutic HSV-2 vaccine

**DOI:** 10.1172/jci.insight.179010

**Published:** 2024-06-18

**Authors:** Emily S. Ford, Alvason Z. Li, Kerry J. Laing, Lichun Dong, Kurt Diem, Lichen Jing, Koshlan Mayer-Blackwell, Krithi Basu, Mariliis Ott, Jim Tartaglia, Sanjay Gurunathan, Jack L. Reid, Matyas Ecsedi, Aude G. Chapuis, Meei-Li Huang, Amalia S. Magaret, Christine Johnston, Jia Zhu, David M. Koelle, Lawrence Corey

**Affiliations:** 1 Vaccine and Infectious Disease Division, Fred Hutch Cancer Center, Seattle, Washington, USA.; 2 Division of Allergy and Infectious Diseases, Department of Medicine, and; 3 Department of Laboratory Medicine and Pathology, University of Washington, Seattle, Washington, USA.; 4 Sanofi, Swiftwater, Pennsylvania, USA.; 5 Translational Sciences and Therapeutics Division, Fred Hutch Cancer Center, Seattle, Washington, USA.; 6 Institute of Stem Cell and Regenerative Medicine and; 7 Department of Global Health, University of Washington, Seattle, Washington, USA.; 8 Benaroya Research Institute, Seattle, Washington, USA.

**Keywords:** Vaccines, Virology, Adaptive immunity, Cellular immune response, T cell receptor

## Abstract

The skin at the site of HSV-2 reactivation is enriched for HSV-2–specific T cells. To evaluate whether an immunotherapeutic vaccine could elicit skin-based memory T cells, we studied skin biopsies and HSV-2–reactive CD4^+^ T cells from PBMCs by T cell receptor (TCR) β chain (*TRB*) sequencing before and after vaccination with a replication-incompetent whole-virus HSV-2 vaccine candidate (HSV529). The representation of HSV-2–reactive CD4^+^ *TRB* sequences from PBMCs in the skin *TRB* repertoire increased after the first vaccine dose. We found sustained expansion after vaccination of unique, skin-based T cell clonotypes that were not detected in HSV-2–reactive CD4^+^ T cells isolated from PBMCs. In one participant, a switch in immunodominance occurred with the emergence of a TCR αβ pair after vaccination that was not detected in blood. This TCRαβ was shown to be HSV-2 reactive by expression of a synthetic TCR in a Jurkat-based NR4A1 reporter system. The skin in areas of HSV-2 reactivation possessed an oligoclonal *TRB* repertoire that was distinct from the circulation. Defining the influence of therapeutic vaccination on the HSV-2–specific *TRB* repertoire requires tissue-based evaluation.

## Abstract 2

T cell receptor B sequencing reveals that HSV-2–specific T cell clonotypes in skin are minimally reflected through PBMC sampling.

## Introduction

While antivirals for HSV-2 have been in widespread use for over 35 years, HSV-2 infection continues to be highly prevalent globally (1–3). As such, development of novel methods to curtail HSV-2 reactivation and transmission are needed. T cell immune responses are associated with severity and reactivation frequency of mucosal HSV infections (4, 5), contributing to interest in the development of immunotherapeutic approaches to control HSV-2 reactivation. Although recombinant HSV-2 protein-based vaccines have been tested, and one demonstrated a partial but significant reduction in the rate of subclinical HSV-2 reactivation among seropositive adults (5–8), correlation between humoral or cellular markers of HSV-2 immunogenicity and vaccine response has not been demonstrated (5, 8–11). Likewise, T cell responses in PBMCs were not associated with efficacy in studies of peptide-based vaccines (12, 13). In a phase I study of HSV529, a replication-deficient HSV-2 whole-virus vaccine, 46% of HSV-2–seropositive participants had a detectable increase in IFN-γ expression in CD4^+^ T cells from PBMCs (14). There was minimal (<10%) change in IFN-γ expression from CD8^+^ T cells over the course of the vaccine trial.

Except during acute infection, HSV-specific CD4^+^ T cells comprise only a small percentage of circulating CD4^+^ T cells (15). T cell production of IFN-γ or other cytokines in response to whole virus or peptide-based libraries is typically used to determine HSV specificity or activity (16–19). In a study of 40 HSV-2–seropositive individuals during clinical quiescence, 0.04%–3.77% (median, 0.31%) of CD4^+^ T cells from PBMCs expressed IFN-γ after HSV-2 stimulation in vitro (20). As a surrogate marker of activation, HSV-specific CD4^+^ and CD8^+^ T cells can express CD137 after in vitro exposure to antigen and antigen-presenting cells (APCs) in activation-induced marker assays (21). Cytokine production after cognate antigen exposure remains the gold standard to identify T cells with HSV specificity used in studies of vaccine response.

Data from animal models, corroborated by mathematical modeling of human data and observational human studies, have supported the importance of skin-resident memory T cells in the adaptive immune responses to HSV-2 reactivation (22–27). These cells are distinct from skin-migratory T cells by phenotype, skin ingress and egress markers, and spatial localization (27–29). A potential explanation for the lack of association between clinical benefit and measured immune response in immunotherapeutic vaccine trials is that the immune correlates of efficacy may lie in alteration of the immune responses in the genital or mucosal skin, rather than the response detected in circulating PBMCs.

To investigate whether a 3-dose vaccination series with HSV529 could elicit changes in the skin-based T cell response, we performed T cell receptor (TCR) β chain (*TRB*) repertoire sequencing and quantitation of the number of copies of each unique *TRB* sequence (clonotype) of genital skin biopsies taken during clinical quiescence from the site of previous symptomatic HSV-2 reactivation before and after each dose. Previous work has shown the stability of this repertoire over time (28, 30, 31). The *TRB* repertoire of HSV-2–reactive CD4^+^ T cells from PBMCs was also obtained to allow for extrapolation of probable HSV-2–specificity among skin-identified clonotypes. CD4^+^ T cells were selected for comparison due to their observed expansion in the phase I clinical trial preceding this study (Clinicaltrials.gov NCT01915212) (14). We found that an immunotherapeutic vaccination elicits skin-based immune responses, including expansion of HSV-2–specific T cell clonotypes. Expansion in HSV-2–specific CD4^+^ clonotypes targeting many varied HSV-2 epitopes was observed in blood. We also found sustained expansion after vaccination of unique, skin-based T cell clonotypes that were not detected in HSV-2–reactive CD4^+^ T cells isolated from PBMCs.

## Results

### Vaccination does not alter T cell abundance or localization at sites of HSV-2 reactivation.

**Figure 1:**
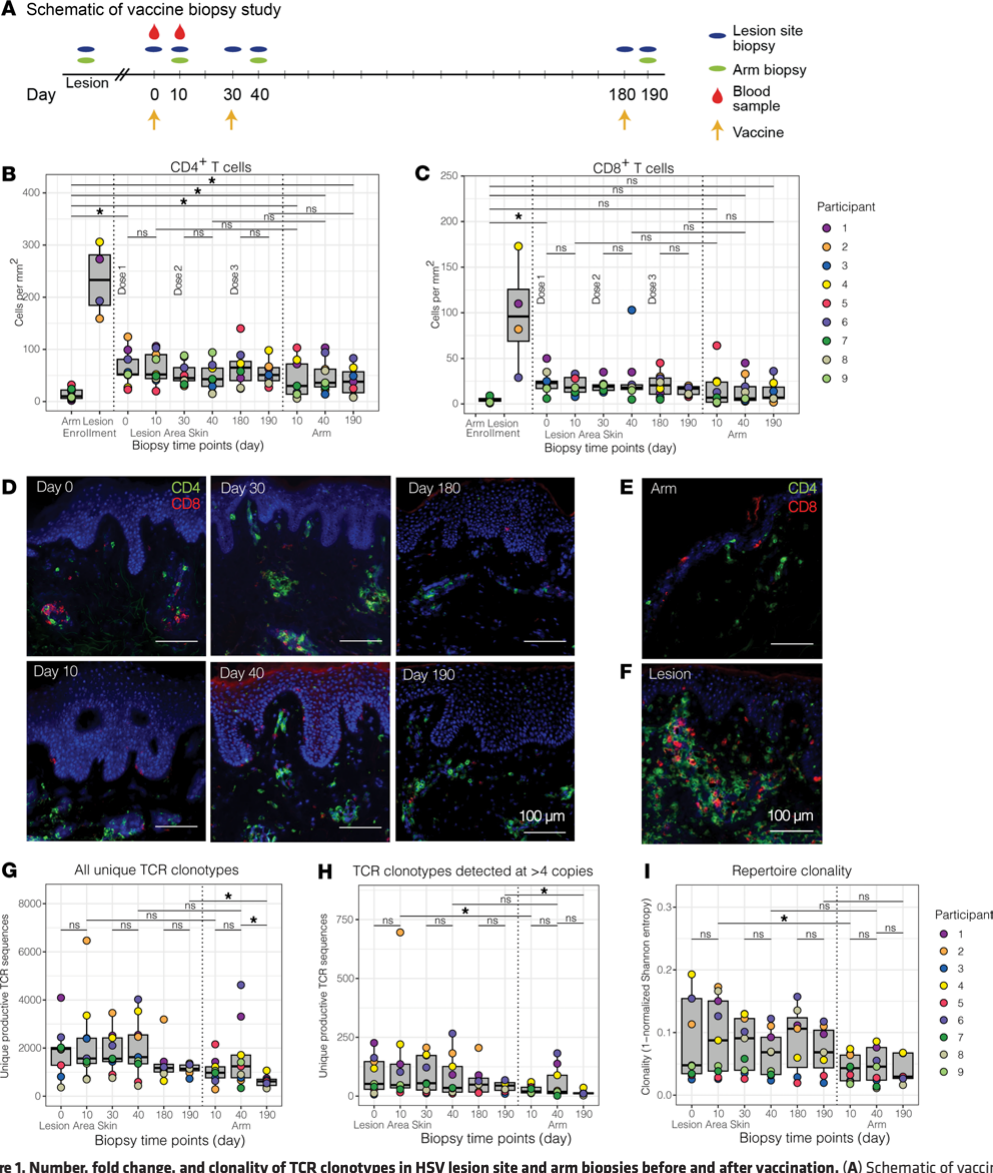
Number, fold change, and clonality of TCR clonotypes in HSV lesion site and arm biopsies before and after vaccination. (**A**) Schematic of vaccine study timeline and procedures. (**B**) CD4^+^ and (**C**) CD8^+^ T cell densities of biopsies from control skin at the time of enrollment and from the site of a symptomatic lesion (*N* = 4) are shown in comparison to HSV lesion site and control skin biopsies over the course of a 3-dose vaccine trial in 9 vaccine recipients. Each dot represents the mean of 3 counted sections in a single participant. Median and interquartile range are shown in gray. (**D**) Representative micrographs (original magnification, ×10) of CD4^+^ (green) and CD8^+^ (red) T cell density by immunofluorescence (IF) in HSV lesion site biopsies at specified time points before and after vaccination. CD4^+^ and CD8^+^ T cell IF from (**E**) control skin and (**F**) lesion site during a symptomatic HSV-2 outbreak. All images are from P4. Scale bars: 100 mm. (**G**) Total and (**H**) number of TCRβ clonotypes detected at >4 copies are shown from the HSV lesion site and arm biopsies. (**I**) Clonality calculated from Shannon entropy of the TCR repertoire from each sample. Each dot represents a single participant. Median and interquartile range are shown in gray. *P < 0.05 by Wilcoxon’s signed-rank test.

To determine whether an immunotherapeutic vaccine against HSV-2 might alter the T cell repertoire at the site of HSV-2 reactivation, 9 persons between the ages of 32 and 54 years, with a known history of symptomatic HSV-2 (median duration, 11.9 years) were enrolled (Figure 1A and Supplemental Tables 1 and 2; supplemental material available online with this article; https://doi.org/10.1172/jci.insight.179010DS1) (Clinicaltrials.gov NCT02571166). Vaccine doses were given at days 0, 30, and 180, and biopsies were performed at days 0, 10, 30, 40, 180, and 190 (Figure 1A). Seven participants completed the entire 3-dose, 7-biopsy study protocol; 2 individuals participated in the full duration of the study but declined biopsies after day 40.

To determine whether vaccination influenced the size of the skin-based T cell repertoire, the density of CD4^+^ and CD8^+^ T cells resident during an active lesion and clinical quiescence before and after each vaccination dose were enumerated in biopsies from the lesion-area skin. T cell immunofluorescence showed that vaccination was not associated with an increase in CD4^+^ or CD8^+^ T cell density at the lesion site (Figure 1, B and C). We also did not observe differences in CD4^+^ or CD8^+^ T cell special localization at either the lesion site or arm by visual inspection. T cells were seen to be distributed in the upper dermis, dermal epidermal junction, or in clusters, as is typically observed. (Figure 1, D–F) (31, 32). The density of total CD4^+^ and CD8^+^ T cell infiltration was lower in the arm at enrollment compared with the lesion area during clinical quiescence (day 0) (Figure 1, B and C). Although vaccination was delivered to the contralateral arm, the number of CD4^+^ T cells in the arm biopsy was significantly higher after vaccination compared with that before vaccination (14.0 vs. 40.0, 49.8, and 39.6 cells/mm^2^, *P* = 0.02, 0.01, and 0.04, respectively), but the number of CD8^+^ T cells did not change significantly. Four participants had genital HSV-2 recurrences (lesions) in the 3 months before vaccination. All biopsies from these lesions demonstrated the massive lymphocyte infiltration typically associated with symptomatic reactivation (Figure 1F).

### The TRB repertoire in lesion-area skin is larger and more diverse than in the arm.

We performed bulk *TRB* sequencing from skin to visualize the *TRB* repertoire in lesion-area skin in comparison to the arm. *TRB* sequencing from lesion site biopsies taken during clinical quiescence, just before vaccination (day 0), identified a median of 1,966 unique clonotypes and 52 clonotypes identified at >4 copies per participant. Vaccination did not result in a significant increase in the median number of unique clonotypes in the lesion area per participant or the number of unique clonotypes detected at >4 copies (Figure 1, G and H). More clonotypes were detected at >4 copies in HSV lesion sites than in the arm at two of 3 sampled time points (day 10 and 190) (Figure 1H). *TRB* repertoire clonality, defined as the inversion of Shannon entropy (a comparative measure of the number and frequency of unique TRB sequences in each sample) (33), was greater in lesion site than arm biopsies at day 10 (*P* = 0.03) but did not change in the lesion site after any of the 3 vaccinations (Figure 1I).

### Vaccination elicited HSV-2–reactive CD4^+^ T cells in blood.

To obtain *TRB* sequences to perform clonal tracking of a known population of HSV-2–reactive CD4^+^ T cells, HSV-2–reactive CD4^+^ T cells were isolated from PBMCs before and after the first vaccine dose (see Methods). At day 0, a median of 30 unique clonotypes per participant were identified (range, 25–51 clonotypes). After vaccination, the median number of unique HSV-2–reactive clonotypes increased 8-fold to a median of 237 clonotypes per participant (range, 46–1,360 clonotypes/participant, unpaired Wilcoxon’s *P* = 0.002, excluding participant 4 [P4], whose cells underwent ex vivo expansion prior to sequencing) (Supplemental Table 3). Almost all of the 233 HSV-2–reactive clonotypes in PBMCs at day 0 were detected at single copy, whereas at day 10, clonotypes were found at >2 copies in 6 of 9 participants, suggesting that an increase in detectable HSV-2–reactive T cells in the blood after vaccination with HSV529 comes from expansion of relatively low abundance clonotypes. The low copy number of each unique T cell sequence, particularly from day 0, is not unexpected because HSV-2–reactive T cells were not expanded in vitro prior to sequencing (Supplemental Figure 1).

### Almost half of vaccine-elicited HSV-2–reactive T cells from PBMCs detected in skin were present in skin prior to vaccination.

**Figure 2:**
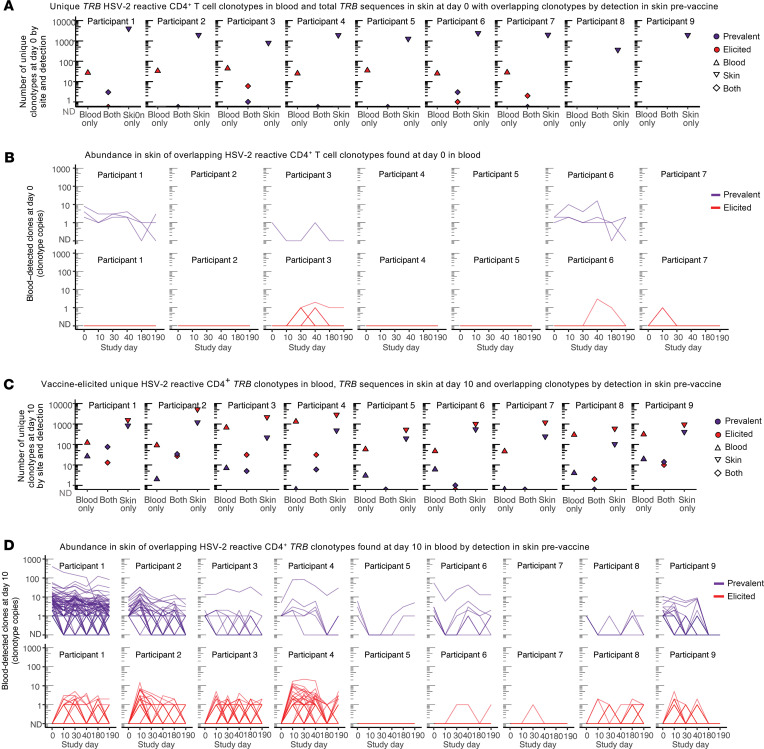
Overlap of HSV-2–reactive CD4^+^ T cells from PBMCs in skin biopsies by clonal tracking before and after initiation of vaccination series. (**A**) Unique *TRB* sequences detected only in blood at day 0 (“blood,” clonotypes from blood at day 0 also detected at any time in skin (“both”), and unique clonotypes in skin at day 0 (“skin”) and (**B**) the longitudinal detection of each of the overlapping (both) clonotypes in lesion-area skin over time, by whether they were observed before or after vaccination. Each line represents a unique clonotype. “ND” is not detected at that time point. (**C**) Unique *TRB* sequences detected in blood and skin at day 10 by detection in one or both sites and (**D**) the longitudinal detection of each of the overlapping clonotypes in lesion-area skin over time. Each line represents a unique clonotype. Shading of both histograms and longitudinal graphing represents whether clonotypes were observed before (purple) or only after (red) vaccination.

We next mapped the frequency of detection and persistence of these PBMC-identified HSV-2–reactive CD4^+^ T cell clonotypes in *TRB* repertoires from lesion-area and control site biopsies. Prevalent clonotypes were defined as clonotypes that had been detected prior to the initiation of the vaccine series, whereas elicited clonotypes were defined as those only detected after initiation of vaccination. Of 205 HSV-2–reactive clonotypes detected in PBMCs from all 7 tested participants at day 0, 16 (7.8%, in 4 participants) HSV-2–reactive blood clonotypes were detected in any skin biopsy from day 0 to day 190, and 7 (in 3 participants) of the 16 (43%) were detected at day 0. Three participants did not have day 0 blood clonotypes detected in skin. Eight clonotypes from blood at day 0 were detected in the active lesion biopsies from 4 participants (Figure 2, A and B, and Supplemental Tables 3 and 4). Ten days after dose 1, 2212 HSV-2–reactive clonotypes were detected in PBMCs; only 5 (0.1%) had also been detected in the day 0 blood sample. 447 of 2,212 (20%) clonotypes were detected in lesion-area skin from day 0 to day 190, with 203 of those 447 (45%) detected in skin at day 0 (from 8 of 9 participants) (Figure 2, C and D; Supplemental Table 3; and Supplemental Table 4, A and B), and 247 of 1,920 had been detected at the time of active lesion in the 4 participants where that biopsy was available (Supplemental Table 4C). Thus, 45% of skin-detected and 9% of all vaccine-elicited clonotypes from PBMCs were present as resident T cells in tissue at study onset (day 0) (Figure 2D). Overlap between the arm at any time point and the day 0 or day 10 HSV-2–reactive clonotypes from blood was low, ranging from 0 to 5 clonotypes per person from the day 0 sample and 0–25 clonotypes per person after vaccination (Supplemental Table 4). By evaluation of characteristic MAIT TRA gene usage (TRAV1-2 and TRAJ families 12, 20, or 33) in P4 and P2, potential MAIT-like cells represented approximately 2% (0.9%–2.6%) of TCR sequences in lesion site and control biopsies and did not change with vaccination (Supplemental Table 5). In summary, vaccination increased the number of detectable, unique HSV-2–reactive CD4^+^ T cell clonotypes in blood (205 to 2,212 clonotypes across all participants). Of vaccine-elicited clonotypes, approximately 20% were detected in skin biopsies at any point, and nearly half of these had been present in genital-area skin at the time of the initiation of the vaccine series.

### Persistence and expansion of skin-based clonotypes.

**Figure 3:**
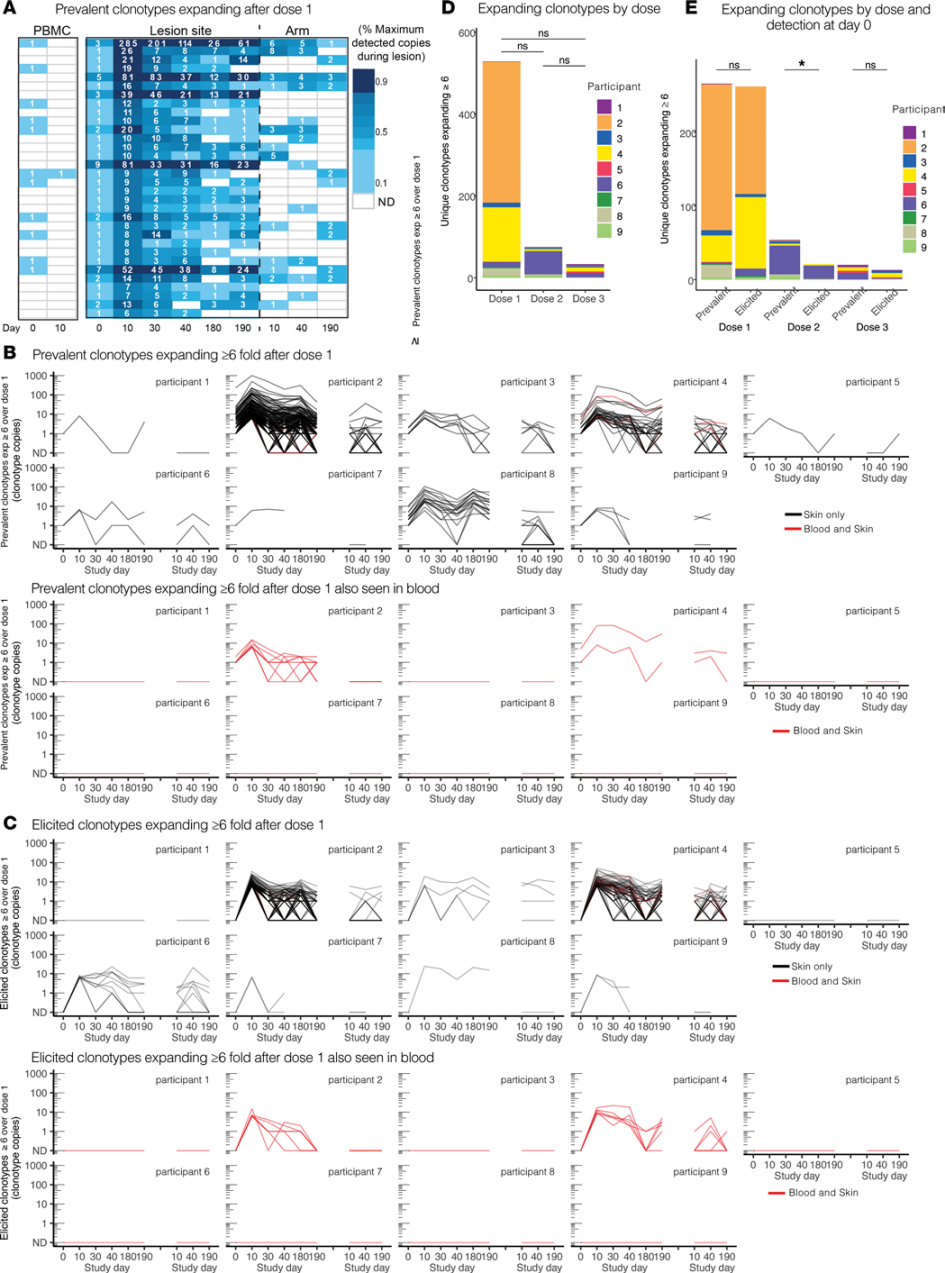
Prevalent and elicited clonotypes in HSV-2–enriched CD4^+^ T cells from PBMCs, healed lesion site, and arm biopsy expanding or detected at high copy number. (**A**) Prevalent clonotypes detected in P4, by fold increase over dose 1. Each row represents a single clonotype (by nucleic acid sequence). Codetection of clonotypes in HSV-2–enriched CD4^+^ T cells from PBMCs, active lesion, quiescent lesion-area skin, and arm skin are compared at each of the time points. The number of copies detected in PBMCs from P4 at day 10 reflects ex vivo expansion prior to sequencing and is denoted in individual cells (see Methods). Blank boxes indicate lack of detection. (**B**) Prevalent and (**C**) elicited clonotypes either expanding ≥6-fold after dose 1 or present after dose 1 (day 10) at ≥6-fold above a single copy and their longevity in lesion-area tissue over the course of the vaccine trial (left), with the corresponding abundance in the arm control (right). ND, not detected. Highly prevalent clonotypes that expanded after the first vaccine dose are seen to persist in tissue throughout the vaccine trial. Color indicates whether the nucleotide sequences were detected in blood (black indicates a tissue-only sequence, red was also seen in blood). Prevalent expanded clonotypes are of greater abundance than elicited clonotypes. Most in-tissue expansion was not from clonotypes observed in blood. (**D**) Stacked bar graphs showing number of clonotypes expanding by ≥6-fold after each dose in the lesion-area skin by person. (**E**) Number of clonotypes expanding by ≥6-fold after each dose in the lesion-area skin by detection prior to initiation of vaccine series (prevalent) versus clonotypes only seen after initiation of vaccination (elicited). *P < 0.05 by Wilcoxon’s signed-rank test.

We next characterized the skin-based repertoire over time to determine how the relative abundance of clonotypes in skin changed over the course of vaccination. To narrow analysis to clonotypes of greatest interest, we identified and ranked the skin-based clonotypes with the greatest expansion (≥6-fold increase in number of copies) after the first vaccine dose (from day 0 to day 10) as “tissue-expanders” and examined their durability in serial biopsies from lesion-area skin, and detection in serial biopsies from the arm and in HSV-2–reactive CD4^+^ T cells from PBMCs. Prevalent clonotypes that increased ≥6-fold after dose 1 at the lesion site were infrequently detected in blood and did not undergo similar expansion in the arm (P4, Figure 3A, all others Supplemental Figure 2). Clonotype trajectories of prevalent clonotypes increasing by ≥6-fold over dose 1 are shown in all participants in Figure 3B. Figure 3C shows elicited clonotypes that were newly identified at 6 copies or greater at day 10 after dose 1. In Figure 3, B and C, clonotypes identified as HSV-2–reactive CD4^+^ T cells from PBMCs are shown in red. The longitudinal detection and CDR3 amino acid sequence of each tissue-expanding clonotype over dose 1 in all participants is shown in Supplemental Figure 2, A and B, and Supplemental Table 6, A and B. The number of prevalent and elicited expanding clonotypes in each participant is summarized in Table 1. Among all participants, there was more expansion (by number of clonotypes increasing by ≥6-fold) in the lesion-area biopsies from day 0 to 10 (median, 11 clonotypes; range, 1–34 clonotypes) than in the arm biopsies from day 10 to 190 (median, 0 clonotypes; range, 0–4 clonotypes; *P* = 0.01 by paired Wilcoxon’s) and day 40 to 190 (median, 0 clonotypes; range, 0–3 clonotypes; *P* = 0.01). Expansion in lesion-area biopsies was greatest after dose 1 and waned after doses 2 and 3, but these differences were not significant by paired Wilcoxon’s (Figure 3D and Supplemental Table 6C). There were a similar number of prevalent and elicited clonotypes expanding after dose 1 and 3 and a greater number of prevalent clonotypes expanding after dose 2 by paired Wilcoxon’s (Figure 3E). Expanding clonotypes represented a greater proportion of prevalent than elicited clonotypes (Table 1). Among 535 prevalent and elicited clonotypes expanding in tissue after dose 1, 21 unique clonotypes were detectable as HSV-2–reactive CD4^+^ T cells in blood from only 2 participants (Figure 3, B and C, and Table 1).

To further characterize prevalent clonotypes and their persistence in tissue over time, we compared their detection in serial biopsies. Prevalent and elicited clonotypes were identified to be resident if they were present in >3 of 5 and >2 of 3 (for those 2 persons who did not have biopsies performed after day 40) after day 0. A median of 9.3% of prevalent clonotypes were resident in tissue after day 0, compared with 1.3% of elicited clonotypes (*P* = 0.004) (Supplemental Figure 2, A and B, and Table 1). Of expanders, a median of 80% of prevalent clonotypes compared with a median of 33% elicited clonotypes were resident after vaccination (*P* = 0.004 by paired Wilcoxon’s). In all, prevalent clonotypes were more persistent in tissue than vaccine-elicited clonotypes.

We compared persistence and expansion over vaccine doses 2 and 3. Supplemental Figure 2C displays the clonotypes that expanded ≥6-fold from day 30 to day 40 before and after dose 2 or were detected at >6 copies at day 40 after not being present prior to vaccination**.** Over all participants, more expansion was detected after dose 1 than over the whole 6-month biopsy series or over the 10 days before and after doses 2 and 3 (Figure 3D, summary in Table 1). P6 was the only participant in whom greater expansion was detected after dose 2 than dose 1. In all participants, 295 clonotypes increased in copy number after dose 2, and more prevalent clonotypes expanded than did elicited clonotypes (Figure 3E). Of those, 72 expanded ≥6-fold over the first dose and 11 expanded ≥6-fold-over the second (day 30 to day 40); only 1 clonotype (in P6) expanded ≥6-fold over both doses.

### Disparity between skin and PBMC clonotypes.

Next, we aimed to quantify the representation of skin-based clonotypes and HSV-2–reactive CD4 *TRB* sequences from blood. *TRB* repertoire sequencing demonstrated that the T cell populations in the skin and blood shared few clonotypes. We analyzed the unique *TRB* sequences identified as clonotypes of interest in skin: (a) those expanding ≥6-fold after the first vaccine dose (*n* = 266), (b) those appearing at ≥6 copies after the first vaccine dose (*n* = 262), and (c) those that were detected in more than 70% of all lesion-area biopsies (*n* = 1,904 total, or 212 per participant on average) (Table 1). Across all samples, 0.7% of skin-based TRB sequences were detected among HSV-2–reactive CD4^+^ T cells from PBMCs (495 unique nucleotide sequences), which reflects 13% of PBMC-detected HSV-2–reactive CD4^+^ T cells (Supplemental Tables 3 and 4). The day 10 blood sample captured 4% of expanded (increased by ≥6-fold after dose 1) or highly elicited (detected at ≥6 copies at day 10) tissue-based clonotypes (22 of 528): 10 prevalent and 12 elicited clonotypes. It also captured 6.0% (114 of 1,904) of clonotypes resident in skin, i.e., clonotypes detected in most skin biopsies after day 0 (either ≥4 of 5 or 3 of 3 for those persons with only 4 biopsies). The blood sample at day 0 did not capture any clonotypes expanded or elicited at ≥6 copies after vaccine dose 1 and captured only 7 (0.2%) of skin-resident clonotypes (Supplemental Tables 3 and 4).

### Asymptomatic HSV shedding was common and unchanged over periods of observation.

HSV-2 shedding (as defined by detection of HSV-2 DNA by PCR on a genital-area self-swab) during the trial was common, occurring in 5 of 9 participants during the first dose (from day 0 to 10), 3 of 9 during the second (from day 30 to 40), and 4 of 9 during the third (from day 180 to 190). All but 2 participants experienced shedding between dose 1 and 2. Persons with or without shedding were not observed to have alterations in their TRB repertoire or greater overlap with HSV-2–reactive CD4^+^ T cells from blood if shedding was situated prior to a biopsy (Supplemental Table 7).

### Stability in V-J usage in skin and shifts in oligodominance after vaccination.

**Figure 4:**
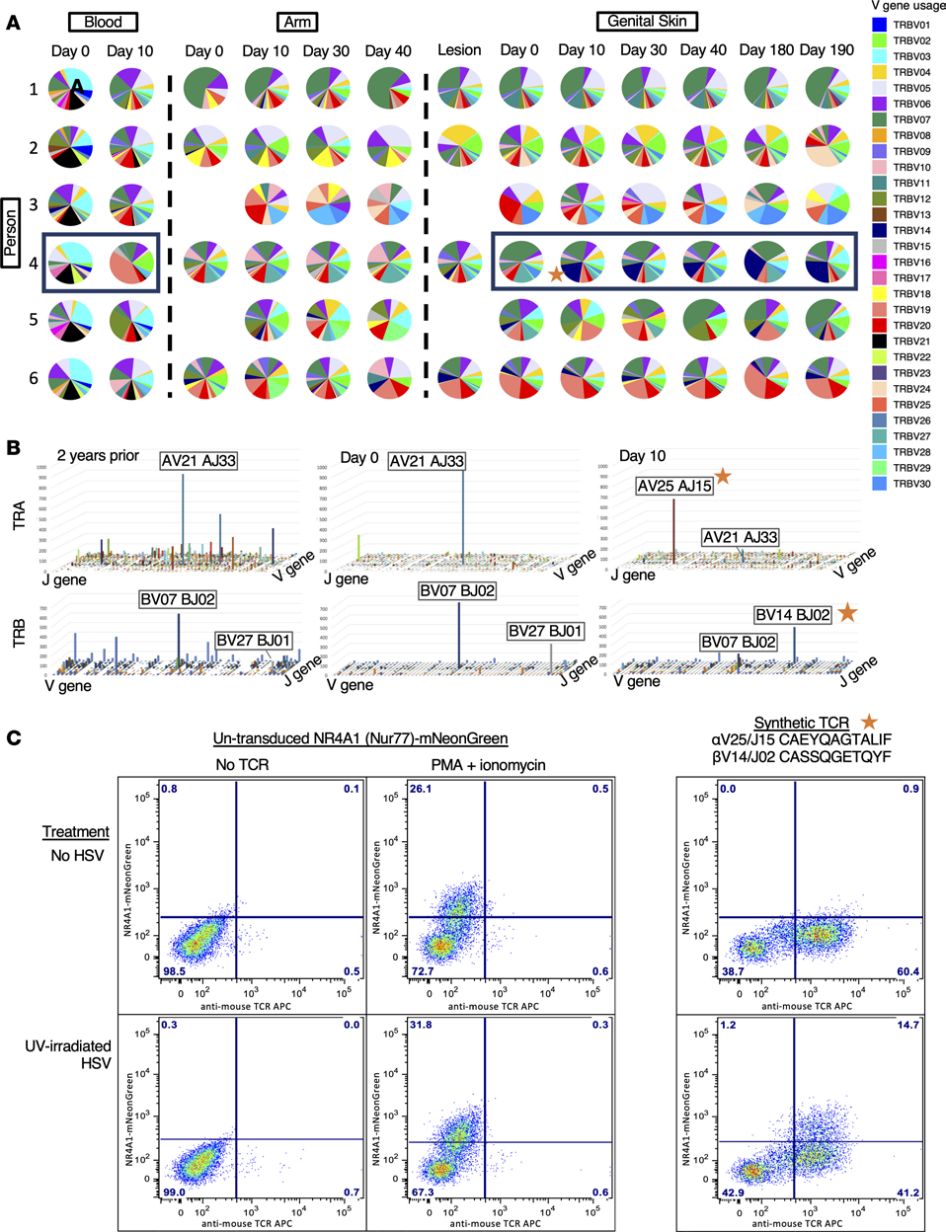
Evaluation of TRA/TRB V and J gene usage. (**A**) V gene usage in participants 1–6 across all sites, including blood, arm, and genital skin, over time. Pie charts demonstrate the proportion of the total repertoire represented by each gene. Skin site clonotypes were limited to those that were present at greater than 4 copies; all HSV-reactive CD4^+^ T cells from blood are shown. (**B**) Combination V and J gene usage in P4 showing the shift in immunodominance from 2 years prior to vaccination through 10 days after the first vaccine dose. The *z* axis represents numbers of copies in each combination. The *x* and *y* axes represent V and J genes listed sequentially. The V and J genes are labeled for the most abundant combinations. (**C**) HSV-2 specificity of a synthetic TCR composed of the most abundant TRA and TRB sequences from the day 10 sample in P4, using a Jurkat Nur77-mNeonGreen reporter cell system (right). Negative control (no TCR, left) and positive control (PMA + ionomycin, middle) for TCR stimulation and negative treatment (no HSV, top) or experimental treatment (UV-irradiated HSV, bottom) to assess TCR specificity. The star denotes the newly immunodominant TCR stimulated by vaccine in P4 (**A** and **B**) and shown to be HSV specific (**C**).

To further investigate changes in *TRB* repertoires after vaccination, we evaluated the distribution of V-J usage within each participant’s *TRB* repertoire. In HSV-2–reactive CD4^+^ T cells from blood, V-J usage changed relatively dramatically between day 0 and day 10. By contrast, V-J usage in the arm was relatively constant across the vaccination series (Figure 4A and Supplemental Figure 3). In genital-area skin, vaccination resulted in a detectable shift of V-J usage in 3 of the 9 participants after the first dose (P3, P4, and P5). In P3, BV24 and BV28 clonotypes were elicited and persisted through day 190. In P5, a BV18 clonotype was elicited at day 10 but was not detectable after day 30 (Figure 4A and Supplemental Figure 3). In P4, TRA/TRB sequencing of archived samples showed that prior to vaccination, a highly oligodominant V-J combination had been dominant for more than 2 years and a previously infrequent oligodominant V-J combination became dominant at day 10. Prior to vaccination (day 0 and in 5 biopsies from 2 years before the vaccine trial) in P4, the most dominant V-J gene combination was TRAV21-01/TRAJ33-01 and TRBV07-09/TRBJ02-01. After vaccination, oligodominance switched to TRAV25-01/TRAJ15-01 and TRBV14-01/TRBJ02-05. Figure 4B illustrates the oligoclonal pattern by TRAV/J and TRBV/J genes of the most prevalent TCR clonotypes 2 years prior to vaccination and at day 0 and day 10 after vaccination in genital-area skin biopsies from P4. This vaccine-shifted, oligodominant V-J combination remained dominant in all subsequent biopsies, through day 190.

We next wanted to determine whether the most abundant postvaccination TRA/TRB pair in P4 (TRAV25-01/TRAJ15-01 CAEYQAGTALIF; TRBV14-01/TRBJ02-05 CASSQGETQYF) was HSV-2 specific. Because it was not detected in blood at day 10 by activation-induced marker–based isolation of HSV-2–reactive T cells from PBMCs, a synthetic TCR with this TRA/TRB pair was expressed in a CD4-expressing Jurkat-based cell line with a NR4A1-mNeonGreen reporter system. NR4A1, also known as Nur77, is activated upon binding of the TCR to ligand. The transduced cell line showed reactivity in response to whole HSV-2 presented by autologous APCs, indicating HSV-2 specificity (Figure 4C). Specificity was refined by showing reactivity to a single HSV-2 protein, VP22, encoded by *UL49*, and a single VP22 peptide corresponding to residues 81–93 (ARPRRSASVAGSH) (Supplemental Figure 4, A and B). To determine if this TCR had the characteristics of TCRs used by CD4^+^ T cells, we examined inhibition of activation of the reporter Jurkat line by HLA class II loci–specific blocking antibodies. A monoclonal antibody that blocks HLA-DP, but not those that block HLA-DR or DQ, was able to prevent reporter cell activation (Supplemental Figure 4C). Together with recognition of killed viral antigen, these data support that this TRA/TRB pair originated in a CD4^+^ T cell, indicating that vaccination elicited HSV-specific tissue-based CD4^+^ T cells not detected in PBMCs.

### Mapping HSV-2 specificity of clonotypes detected in both PBMCs and skin biopsies.

**Figure 5:**
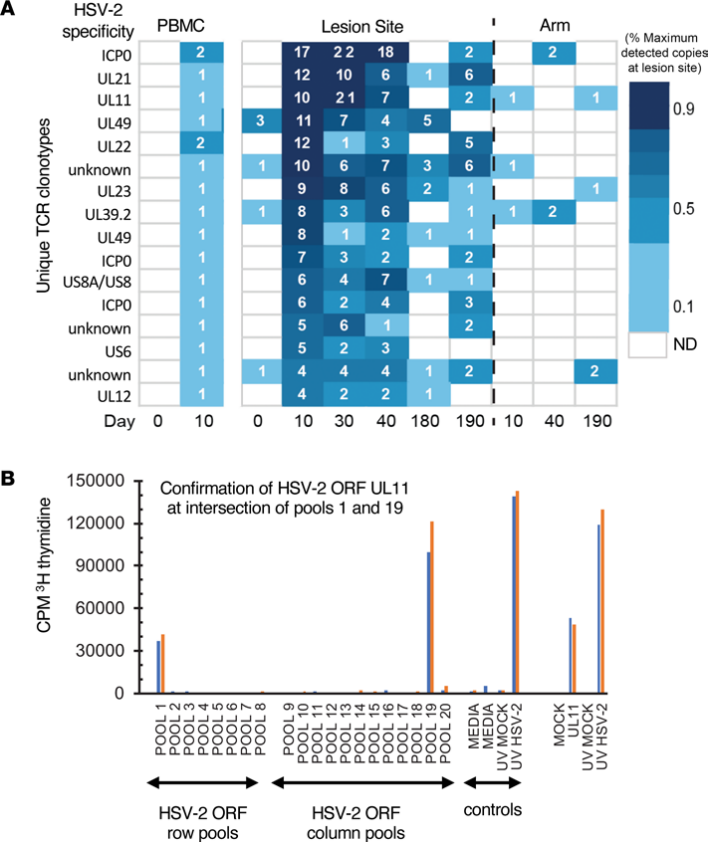
Representative data from fine specificity determination of blood CD4^+^ T cell clones overlapping with TCRβ CDR3 sequences detected in HSV lesion site biopsies. (**A**) Clonotype tracking with assigned specificity of 13 of the 16 clones queried in P4 by abundance and fold change in skin over dose 1 (day 0–10). (**B**) Example of fine-specificity mapping of a single clone confirmed to be specific to UL11. Both specimens were obtained from day 10 after HSV529 vaccination. At left is T cell proliferation in response to matrix pools of HSV-2 antigens with positive and negative controls. Pools containing US11 (pool 1 and pool 19) are positive. At right is confirmatory assay with recombinant UL11 and controls. Blue and orange bars represent 2 replicate assays.

To explore the antigenic specificity of clonotypes that were shared in the blood and skin compartments in P4, we evaluated CD4^+^ T cells reactive to HSV-2 from day 10 PBMCs by single-cell *TRA/TRB* sequencing and fine-specificity determination (Supplemental Figure 5). HSV-2 reactivity was determined by expression of CD137 (4-1BB) by memory T cells after activation through TCR by UV-HSV186, yielding live, antigen-reactive cells for single-cell cloning directly ex vivo (21). Among 192 clones generated from CD4^+^ T cells with high CD137 expression after stimulation with UV-HSV186, 190 proliferated in response to whole HSV-2 antigen, suggesting highly efficient enrichment of HSV-2–reactive clones (Supplemental Figure 5, A and B). Paired *TRA/TRB* sequencing yielded a single productive *TRB* and either 1 or 2 productive *TRA* CDR3 sequences in 160 of these clones (Supplemental Figure 5C and Supplemental Table 8). Of these 160, we selected 16 clones that had perfect *TRB* nucleotide sequence matches in genital skin and had characteristics of clonotypes of interest — either expanding ≥4-fold after the first dose or identified at ≥4 copies at day 10 — and determined their viral protein-level specificity using a library of all curated HSV-2 proteins (30). Figure 5A shows the number of copies detected in skin and blood and TCR specificity from all 16 clones. Reactivity was observed to proteins encoded by genes *ICP0, UL11, UL22, UL23, UL39*, *UL49*, and *US6,* among others, indicating that among these overlapping clonotypes antigenic specificity did not seem to be related to their detection in both compartments (Supplemental Table 9). A representative example of HSV-2 protein UL11 specificity (clonotype 3 in Figure 5A) is shown in Figure 5B. This also confirmed HSV-2 specificity of 16 clonotypes in P4 that were observed to either expand from day 0 to day 10 or were newly detected in tissue after dose 1 at ≥4 copies, all of which were resident in tissue; these clonotypes were identified as clonotypes of interest. Therefore, in this participant, vaccination induced broad CD4^+^ T cell reactivity in the skin at a site of previous HSV-2 reactivation.

Analysis using a sequence-similarity algorithm (TCRdist3) was undertaken to determine the publicity of HSV-2–reactive clonotypes among these 9 participants with chronic HSV infection and their relationship with clonotypes observed to expand. Among all HSV-2–reactive or HSV-2–specific clonotypes from blood and 1,100 total sequences observed to expand by or be elicited at ≥4 copies and using a conservative definition of TRB sequence similarity (<13 sequence-similarity distance units (nearest-neighbor distance), 38 clusters of clonotypes involving 3 or more clonotypes were identified, including 10 with sharing among participants and 11 with sharing among sites (Supplemental Table 10).

## Discussion

Our study reveals several potentially new observations pertinent to immunotherapeutic vaccines or systemic immunotherapies for infectious and, likely also malignant, diseases. Immunization with a recombinant, replication-defective vaccine delivered intramuscularly in the arm elicited expansion of HSV-specific responses in blood and skin near a typical HSV lesion site in all participants. Demonstration of T cell expansion after vaccination was more sensitive through evaluation of skin biopsies than through PBMC sampling. While vaccination led to an increase in detection in blood of clonotypes also resident and highly abundant in skin, these represented a small minority of the total skin clonotypes and clonotypes of interest. *TRB* clonotypes that increased in abundance after vaccination in skin or appeared as vaccine elicited in blood were often from the population of clonotypes present in skin prior to vaccination (both during quiescence at day 0 and/or in an active lesion at enrollment). Our findings are compatible with our current understanding of the immune response to chronic infection with HSV-2: there is a population of skin-resident T cells that is located at the site of reactivation and is poised to respond to viral challenge as it occurs (28, 32, 34). Moreover, among vaccine-elicited and vaccine-expanded clonotypes in skin, clonotypes already present at the time of vaccine initiation were more likely to remain resident in skin. Therefore, for a sustained response, it is tissue-based clonotypes to which immunotherapy must be directed. Unfortunately, a viable and robust mechanism by which to pursue this in humans has not yet been identified. The vaccine we used was discontinued from further study due to the relatively poor immunogenicity based on standard assays; however, the immune responses detected by TRB clonotype tracking were substantially different than predicted by PBMC intracellular cytokine staining. Whether heightened tissue-based responses would be of clinical benefit or whether tissue-based responses might predict clinical efficacy requires study. Novel technologies to recruit and bolster skin-based resident T cells should be a focus for the development of immunotherapeutic vaccines against HSV-2.

The uniqueness and persistence of the skin-resident population are shown most strikingly in P4, in whom TRB repertoire sequencing was possible for samples from 2 years prior to the present study. From these pretrial biopsies, an oligoclonal population (>10% of TRB clonotypes derived from the same V-J combination) was detected in genital region biopsies and was not present in the arm or in HSV-reactive CD4^+^ T cells from PBMCs. A switch in the immunodominant oligoclonal “swarm” of clonotypes, from one dominant V-J combination to another, occurred after the first vaccine dose and was sustained for the full 6 months of this study with a single dominant *TRB*. By creation of a synthetic TCR, we confirmed that this vaccine-elicited, dominant T cell clonotype originated from an HSV-2–reactive CD4^+^ T cell, and its specificity was mapped to UL49.

Over all participants, vaccination, including repeated boosting doses, was not associated with an increase in the total cell population at the site of HSV-2 reactivation, as measured by T cell density, and indeed, boosting did not appear to enhance responses described here after dose 1 by T cell count or clonal tracking. In fact, the expansion response to subsequent doses appeared to diminish from dose 1 to doses 2 and 3. This cannot be explained by the available data presented here, but tissue-based study of subsequent vaccine trials may shed light on whether this may reflect antigen reexposure fatigue or repertoire trimming. In all participants, selective increases in clonotypes were observed and detection of shared HSV-2–reactive TRB sequences increased after the first vaccine dose, albeit expansion varied considerably by participant. Determinants of such variability absolutely require further evaluation. These data indicate that immunogenicity studies of immunotherapeutic vaccines should carefully assess alterations in the TCR repertoire of the pathogen under study and not necessarily a total quantitative increase in antigen-specific T cells.

Within-person *TRB* nucleotide sequence identity with defined HSV-2–reactive clonotypes from PBMCs was used here as a surrogate indicator of HSV-2 specificity of tissue-based TRB sequences. This mechanism undoubtedly misses a large proportion of tissue-based HSV-2–specific T cells, such as the nonblood clonotype shown to be HSV-2 specific when expressed in a reporter system, but the true extent of HSV-2 specificity among tissue-resident T cells has not been defined. Alternative methods to expand the capacity to determine the specificity of tissue-based T cell clonotypes are in development, but clonal tracking is one emerging method to expand the capacity to define T cell specificity into difficult to reach compartments.

The lack of overlap between skin and CD4^+^ T cells from PBMCs is magnified by the ability to select for CD4^+^ T cells with HSV-2 specificity from PBMC samples, which was not feasible for skin biopsies. Indeed, the contribution of CD8^+^ T cell clonotypes to the observed vaccine response in the skin is unknown, and although CD4^+^ T cell expansion was predicted by PBMC-based analysis in initial vaccine trials, this may not be representative of the response in skin. Altogether, even when selecting for those TCR clonotypes in greatest abundance in skin where overlap with PBMCs seems most likely, there was little representation in the HSV-2–enriched CD4^+^ T cell samples. In a chronic viral disease for which an effective vaccine has not been found after many years of trials, we wonder whether the focus on responses in PBMCs may partly explain previous failures. Our findings suggest that the inclusion of skin-based responses in the design of immunotherapeutic vaccines with a skin-based target may provide insights and perhaps bring an effective vaccine for HSV-2 and other chronic infections closer to reality. This study has shown conceptually that therapeutic targeting of T cells resident in genital skin sites by immunotherapeutic vaccination is possible.

## Methods

### Sex as a biological variable.

Men and women were enrolled in this clinical trial. Due to the small study size, results were not stratified by sex but are anticipated to be applicable to both sexes.

### Study design.

This study was designed as an open-label phase I study to observe the shifts in the *TRB* repertoire in the skin at the site of HSV-2 reactivation in comparison to HSV-2–reactive CD4^+^ T cells obtained from peripheral blood and biopsies from an uninvolved arm site over the course of a 6-month, 3-dose vaccination study with replication-incompetent vaccine candidate HSV529 (Sanofi). This study was specifically designed to evaluate whether immunologic changes occurred in sequential genital skin biopsies following HSV529 vaccination. Arm biopsies, lesion-site biopsies during a symptomatic lesion, and lesion-site biopsies following HSV suppression with acyclovir were used in each participant as internal controls — all participants received vaccine (see study schema in Supplemental Table 1 and Figure 1A). Demographic information is shown in Supplemental Table 2.

### Participant recruitment and enrollment.

Healthy men and women with a history of symptomatic genital herpes in areas amenable to biopsy were recruited to the University of Washington Virology Research Clinic in Seattle, Washington, USA. Sex was not considered as a biological variable. HSV-2 seropositivity was confirmed by Western blot (35). Persons living with HIV were excluded.

### Vaccine.

The vaccine contained 0.5 mL (1 × 10^7^ PFU/dose) of HSV529, a replication-deficient, double-deletion (*UL5* and *UL29*) HSV-2 strain (Sanofi) (36). Doses were delivered by intramuscular injection into the deltoid at days 0, 30, and 180. (Figure 1A and Supplemental Table 1).

### HSV lesion-area skin biopsies.

Genital-area skin biopsies (3 mm) were obtained at the site of HSV-2 reactivation, confirmed either by observation of a lesion at a study visit or by reported history. At enrollment, participants had a baseline biopsy performed at the site of a lesion, if present, or at the site of the most frequent recurrence, and received 4 weeks of valacyclovir 500 mg daily to suppress HSV-2 reactivation. Participants stopped valacyclovir 3 days prior to vaccination. Lesion site skin biopsies were performed prior to each vaccine dose on days 0, 30, and 180, and 10 days after each dose at days 10, 40, and 190 (14). Non-HSV-involved skin biopsies were obtained from the arm (contralateral to vaccine dose) at days 0, 10, 40, and 190. Biopsy samples were fresh frozen in optimum cutting temperature compound. Participants performed self-swabbing of the genital area daily for the first 30 study days and for 10 days after each vaccine dose. Detection of HSV DNA by PCR was performed as described previously (37).

### DNA extraction and sequencing.

A 1 × 3 mm cross-section (inclusive of epidermis and dermis) from each biopsy was digested with proteinase-K, and genomic DNA (gDNA) was extracted by spin column (Qiagen DNeasy). *TRB* repertoire sequencing was performed using gDNA samples by Adaptive Biotechnologies using the ImmunoSEQ assay (38). TCRα (*TRA*) chain sequencing (Adaptive Biotechnologies) of the same gDNA samples was performed for 2 participants. Adaptive TCR sequencing is a gDNA PCR-based platform in which the number of *TRB* sequence reads is proportional to the number of T cells bearing that unique sequence in each sample (39). In our experience, a variable but small proportion of HSV-2–specific T cell clonotypes with the same *TRB* nucleotide sequence will be paired with different *TRA* sequences (40). The number of unique *TRB* sequences (including *CDR3*, V, D, and J chain regions) is therefore a surrogate for the number of cells of that clonotype. *CDR3* nucleotide sequences plus V and J chain region identifications were used to compare persistence and detection of a specific clonotype within samples from a single participant. CDR3 amino acid sequences (with V and J chain usage) were used to compare clonotype publicity across multiple participants.

### Isolation of PBMCs.

In the initial trial of HSV529, the majority of alterations in HSV-2–specific T cell responses in PBMCs from HSV-2–seropositive participants were in the CD4^+^ T cell population (14). HSV-2–reactive CD4^+^ T cells were enriched from PBMCs obtained at days 0 and 10 by two methods. In the first of these, PBMCs were incubated with UV-inactivated cell-associated HSV-2 strain 186 (UV-HSV186) for 18 hours and stained for IFN-γ and IL-2 by intracellular staining (as modified from Moss et al*.*, ref. 20). Cytokine-producing cells were sorted, DNA was prepared with the Qiagen blood kit, and *TRB* sequencing was performed as above (Supplemental Figure 1). In 1 participant (P4), at day 10, two selection procedures were performed iteratively to enhance the detection of HSV-2–reactive T cells from PBMCs. After incubation with UV-HSV186, CD3^+^CD4^+^CD8^–^CD137^hi^ cells were sorted and expanded polyclonally (as modified from Jing et al*.*, ref. 21), stimulated again with UV-HSV186, and stained for IFN-γ and IL-2 by intracellular staining (Supplemental Figure 1, method 2). gDNA extraction and *TRB* sequencing of bulk cytokine-producing cells was performed as above.

### Confirmation of HSV-2 specificity and determination of target epitope in PBMC-based clonotypes.

Live CD3^+^CD4^+^CD137^+^ cells (*n* = 960) from frozen PBMCs obtained from P4 at day 10 after dose 1 were single-cell sorted and cloned as published previously (16). We selected 192 random clones for functional screening with autologous irradiated PBMCs as APCs, UV-HSV186 or UV-mock virus control as antigens, and tritiated thymidine incorporation proliferation assay to determine T cell proliferation in response to the given stimuli. We performed paired *TRA* and *TRB* *CDR3* sequencing on aliquots of approximately 100 cells per clone using a SMARTSeq-2 cDNA procedure (41). The specificity of selected clones was determined to the level of the antigenic HSV-2 open reading frame as previously described (30). Briefly, each HSV-2 ORF was cloned and expressed in vitro. T cell clones were expanded once as described previously (17). HSV-2 proteins were queried in proliferation assays as pools of 8–12 proteins/pool in matrix array at final concentrations of approximately 1:5,000 of each protein. To confirm specificity, HSV-2 protein(s) from pools at the intersection(s) of positive rows and columns were employed as antigens in subsequent proliferation assays.

### Development of reporter cell line.

To create the NR4A1_NeonGreen TCR reporter, the coding sequence of mNeonGreen was integrated in-frame into the *NR4A1* locus before the stop codon using CRISPR-induced homology directed repair (42). The mNeonGreen coding sequence (43) (IDT) flanked by a 5′ 334 bp homology arm, 5′ T2A element, and a 315 bp 3’ homology arm was electroporated with recombinant spCas9 (IDT) and *NR4A1* CRISPR guide RNA (IDT, sequence AUGAAGAUCUUGUCAAUGAU) into Jurkat clone E6-1 cells (ATCC). Cells were cloned by limiting dilution, and the clone with the highest signal upon stimulation with PMA (5 ng/mL) and ionomycin (5 μg/mL) (Invivogen) was identified. The obtained reporter cell line was further modified by CRISPR knockout of *TRA*/*TRB* expression (IDT, gRNA sequences AGAGUCUCUCAGCUGGUACA, CAAACACAGCGACCUUGGGU); cells with successful knockout were sorted for lack of CD3 expression. The final NR4A1_NeonGreen TCR reporter showed upregulation of the reporter signal according to TCR signal strength, mimicking regulation of the endogenous *NR4A1* locus (44).

### Creation and HSV-2 specificity testing of synthetic TCR.

Full-length, codon-optimized, TCR genes were synthesized (IDT) with *TRB* preceding a P2A sequence. *TRA/TRB* pairing was promoted by partially replacing portions of the *TRA* and *TRB* constant regions with murine homologs with added cysteine residues. An epitope on the extracellular domain of murine *TRB* enabled flow cytometry monitoring of transduction as previously reported (45). The TCR inserts were cloned into pRRLSIN.cPPT.MSCV/GFP.WPRE (46) via Gibson assembly (New England Biolabs). After confirmation by sequencing, lentivirus was produced using a third-generation system (Addgene) on LentiX-293T cells (Takada Bio) (45). Sequences are available from GenBank (MZ821077–MZ821078).

### Specificity assay.

200,000 synthetic TCR-transduced reporter cells were cocultured with 200,000 participant-matched PBMCs and 1:100 dilution of UV-HSV186 virus or mock for 16–20 hours. Untransduced and PMA/ionomycin-activated reporter cells were used as controls. Cells were labeled with Live Dead Aqua (Invitrogen) and anti-mouse TCR APC (BD Pharmingen) and analyzed on a FACS Canto II for expression of mNeongreen.

### Protein-level specificity assay.

Protein-level specificity was assigned by measuring mNeongreen expression after 18-hour incubation of TCR-transduced reporter cells with matrix-pooled protein antigens (as above) spanning the HSV-2 proteome. Protein-level specificity was confirmed by a comparison between the HSV-2 protein of interest and a nonreacting protein at a range of concentrations (1:400 to 1:1600) for 18–24 hours.

### Peptide-level specificity assay.

Peptide-level specificity was assigned by exposing the TCR-transduced reporter cells to pooled, linear peptides (1 μg/mL) covering the UL49 sequence.

### Blocking assay.

Monoclonal antibodies against HLA-DP, DQ, and DR at a 1:40 final dilution were added to the specificity assays above, in which UV-HSV186 was also serially diluted (1:100, 1:1,000) to determine HLA specificity (16).

### Immunofluorescence staining.

To evaluate the effects of vaccination on T cell infiltration, we evaluated T cell spatial localization and skin density as previously described (32). Thawed, 8 μm skin sections were washed, fixed in acetone, blocked with 1× casein (Vector Laboratories), 2% bovine serum albumin, and 5% normal human sera; incubated for 1 hour in blocking solution at room temperature with mouse anti-human CD4 (1:500; Biolegend); and tagged with Alexa Fluor 488 by tyramide signal amplification (TSA) (Invitrogen), followed by staining with Alexa Fluor 647–labeled mouse anti–human CD8^+^ (1:100; Caltag Laboratories) overnight at 4°C. After nuclear staining with DAPI (Fluka), skin sections were mounted in Mowiol 40-88 containing 2.5% wt/vol DABCO (Sigma-Aldrich). Images were captured with Nikon Eclipse Ti with NIS-Elements AR Software (v4.40) and viewed in Fiji v2.9 (47). CD8^+^ and CD4^+^ T cells were counted manually (in triplicate) in fields of 650 μm^2^, including the epidermis, dermal-epidermal junction, and the upper dermis (48). To evaluate whether vaccination induced an upregulation of cell surface markers associated with tissue homing and residency, immunofluorescence with an antibody combination of mouse anti-human CD4, followed by TSA amplification, Alexa Fluor 647–labeled mouse anti-human CD69 (1:50; Biolegend) and rabbit anti-human CXCR3 (1:50, Abcam) with Alexa Fluor 546 donkey anti-rabbit secondary (1:100, Invitrogen) was performed, as above.

### Statistics.

Comparison of T cell count and density in tissue as well as number of unique *TRB* sequences (clonotypes) between time points, cell populations, and blood/biopsy sites was performed by Wilcoxon’s signed-rank tests where paired comparisons were used for time points where all participants contributed blood or biopsies, and unpaired comparisons were used for comparisons across day 180 and day 190 biopsies (where only 7 participants contributed biopsies) or day 0 in blood (where 7 persons contributed blood). For comparison of T cell count in tissue, the postvaccination lesion-area biopsies were tested against the arm control and the day 0 baseline biopsy specimens. For comparison of clonotype abundance, lesion-area biopsy time points were compared against corresponding arm biopsy time points (10, 40, and 190 days after dose 1). To compute fold change in abundance over time when clonotypes were not present at both time points, a minimum abundance level of 1 copy was assigned to absent clonotypes. *TRA/TRB* sequence data were exported from the Adaptive Biotechnologies platform and analyzed off-line. Analyses were performed using SAS 9.4 (SAS Institute), Python 3.6.5, and R 3.4.1 (R Core Team). Nonproductive sequences and those without V and J region identification were excluded from analysis. Statistical significance was defined as a 2-sided *P* ≤ 0.05. Sequence similarity was evaluated for unique CDR3 amino acid sequences from blood (limited to clonotypes observed at >1 copy from the sample that underwent ex vivo expansion prior to sequencing) and those expanding after vaccination in skin using TCRdist3 (v0.2.2) (49, 50). Clonotypes considered to be similar were restricted to those within 13 nearest-neighbor distance units, which would allow up to 4 exchanges of biochemically similar amino acids or 1 biochemically dissimilar amino acid or deletion in the CDR3 region or up to 12 exchanges in other regions. Logoplots were created with ggseqlogo.

### Study approval.

Participants provided informed consent to participate in the trial. The University of Washington Human Subjects Division approved the study procedures. The trial was monitored by a Data Safety Monitoring Board. The trial was registered on Clinicaltrials.gov (NCT02571166).

### Data availability.

Data used to produce each figure are available in the Supporting Data Values file. The TCR sequencing data that support the findings of this study are available for review at Adaptive Biotechnologies (https://clients.adaptivebiotech.com/pub/ford-2024-jci). The custom code used for these analyses is available at GitHub (https://github.com/esford3/HSV529_manuscript and https://github.com/alvason/visualizing_Tcell_response_after_HSV2_vaccine) or by reasonable request from DMK or LC.

## Author contributions

CJ, ASM, JZ, and LC designed the study. ESF, AZL, LJ, ASM, KMB, DMK, and LC provided TCR data analysis and bioinformatics. LD, LJ, KJL, KB, MO, and DMK provided PBMC-based T cell assays and analysis. ESF, MLH, and JZ analyzed skin biopsies. KD provided synthetic TCR specificity. ME, JLR, and AGC designed the reporter cell line to test TCR specificity. CJ recruited participants and provided clinical guidance. ESF, ASM, KMB, and LC provided statistical analysis. ASM provided statistical consulting. JT, SG, and LC acquired vaccine and provided regulatory guidance. ESF, LC, KJL, DMK, CJ, and ASM prepared the manuscript. All authors reviewed and approved of the manuscript.

## Supplementary Material

Supplemental data

Supplemental tables 1-10

Supporting data values

## Figures and Tables

**Table 1:**
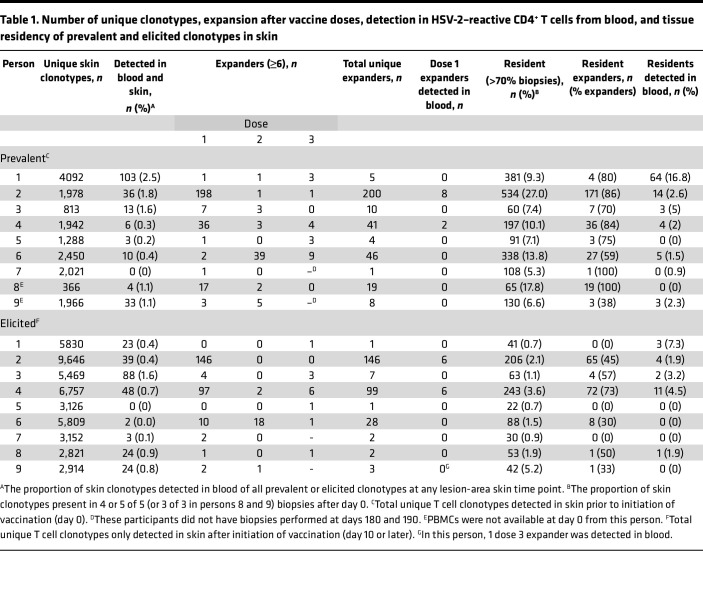
Number of unique clonotypes, expansion after vaccine doses, detection in HSV-2–reactive CD4^+^ T cells from blood, and tissue residency of prevalent and elicited clonotypes in skin

## Version 1 07/22/2024

Electronic publication
