# Supplementary material for: Expansion of the HSV-2–specific T cell repertoire in skin after immunotherapeutic HSV-2 vaccine
Source: JCI Insight. 2024 Jun 18;9(14):e179010. doi: 10.1172/jci.insight.179010 (PMC11383358; doi:10.1172/jci.insight.179010)

Supplementary figures and tables

6.3.24

Supplementary Figures:

Supplemental Figure 1. Experimental procedure to isolate HSV-reactive CD4+ T cells.
Supplemental Figure 2. All prevalent and elicited dose 1 and dose 2 expanding clonotypes from skin and cross-detection in blood and arm.
Supplemental Figure 3. TRBV and TRBJ gene usage in blood, arm, and genital skin.
Supplemental Figure 4. Determination of UL-49 reactivity and HLA-DP restriction of transgenic, vaccine-expanded immunodominant TCR from P4.
Supplemental Figure 5. Gating strategy and CMP results to identify HSV-specificity of clonotypes.

Supplementary Tables:

Supplemental Table 1. Procedures and study visits.
Supplemental Table 2. Demographic characteristics of participants enrolled in the vaccine and preliminary arms.
Supplemental Table 3. Unique clonotypes identified as HSV-2-reactive in CD4+ T cells from blood and their detection in tissue.
Supplemental Table 4a. All prevalent clonotypes detected in blood and skin.
Supplemental Table 4b. All elicited clonotypes detected in blood and skin.
Supplemental Table 5. TRAV/TRAJ usage suggestive of MAIT cell derivation represent a small minority of the TCRa clonotypes detected in two participants over time.
Supplemental Table 6a. All prevalent clonotypes expanding over dose 1.
Supplemental Table 6b. All elicited clonotypes expanding over dose 1.
Supplemental Table 6c. All clonotypes expanding over dose 2.
Supplemental Table 7. Detection of HSV-2 by genital-area swab (viral shedding) by study day.
Supplemental Table 8. All HSV-specific clonotypes from P4.
Supplemental Table 9. Paired HSV-specific TCR sequences with antigen specificity from P4.
Supplemental Table 10. Sequence similar TCR clusters

Supplementary Figure 1.

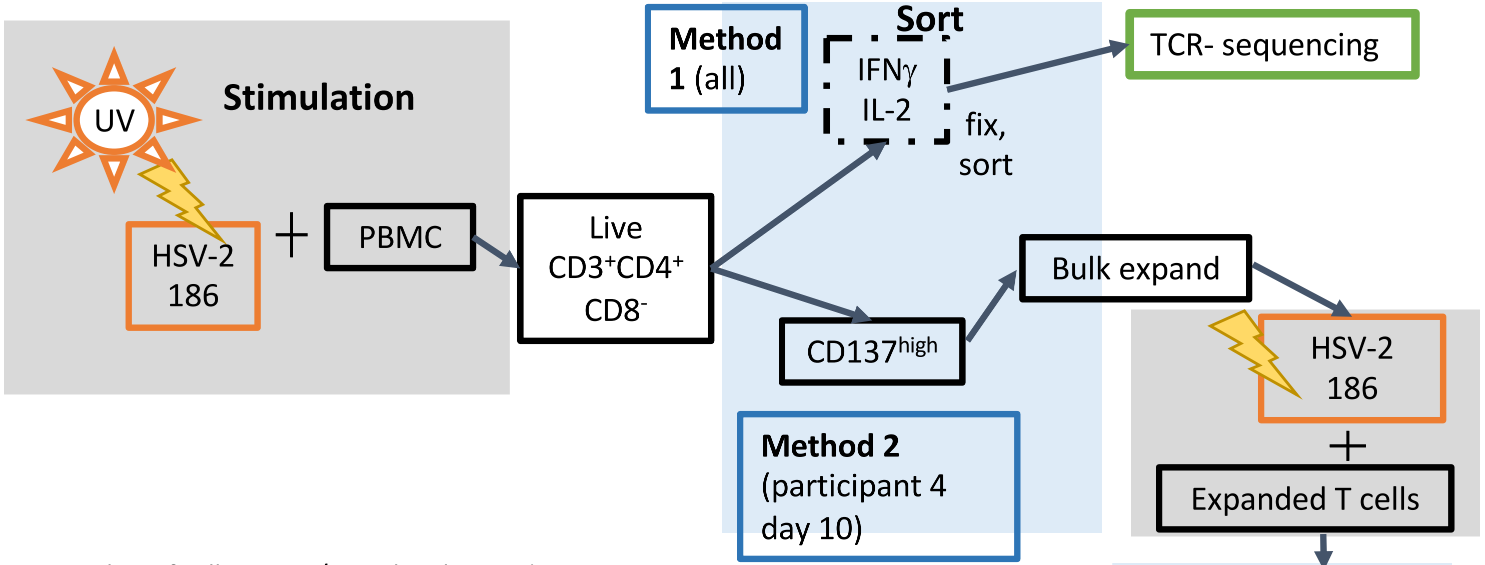

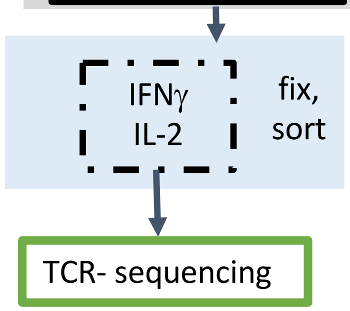

Number of cells in ICS -/+ pools submitted to sequencing

Participant	Day 0 – neg pool	Day 0 submitted	Day 10 – neg pool	Day 10 submitted
1	289082	642	401533	2063
2	120855	768	360478	1478
3	386782	1032	440487	9688
4	297566	7696		
5	264471	626	95977	430
6	142644	165	107899	403
7	238454	125	124473	343
8	NA	NA	451953	13161
9	NA	NA	314766	10686

Example – Method 1
ICS fix/sort, Participant 2

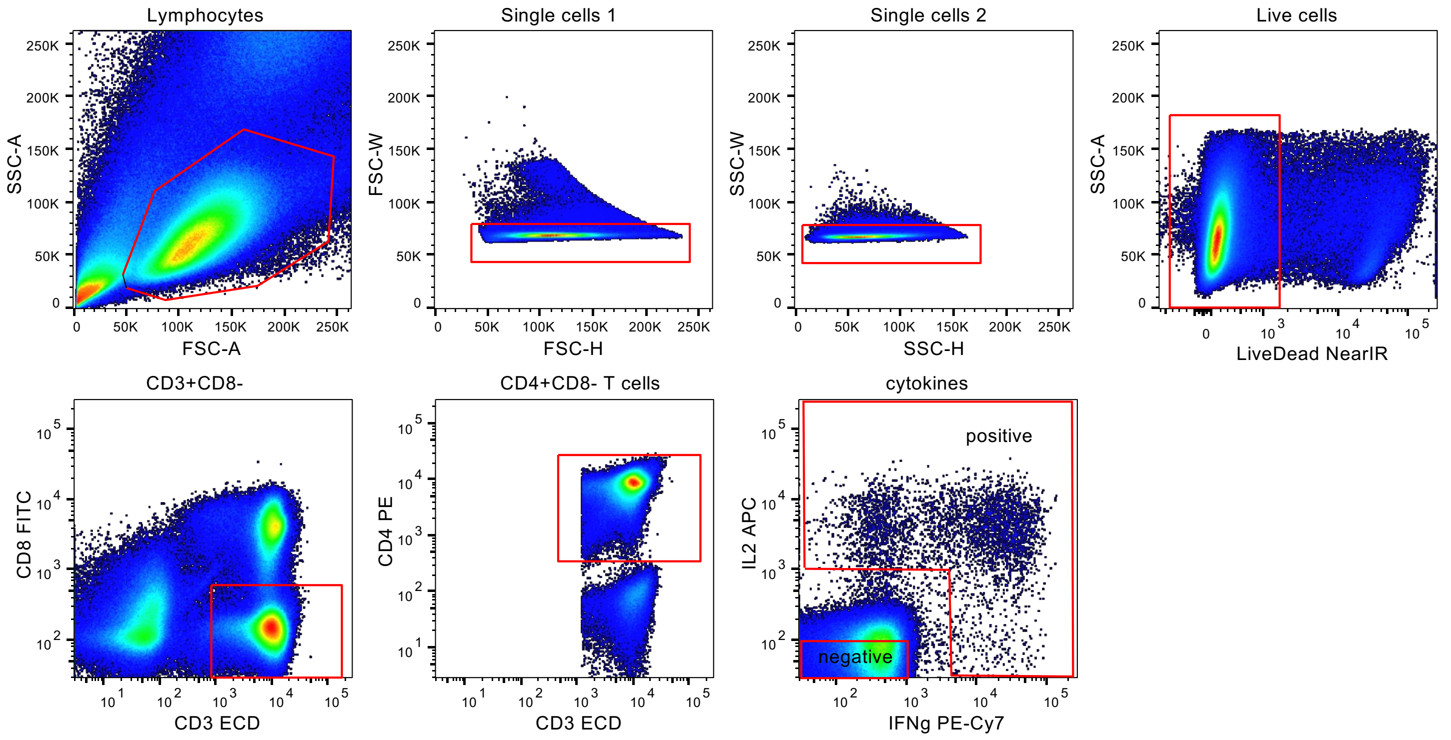

Supplementary Figure 2. All prevalent and elicited clonotypes expanding over dose 1

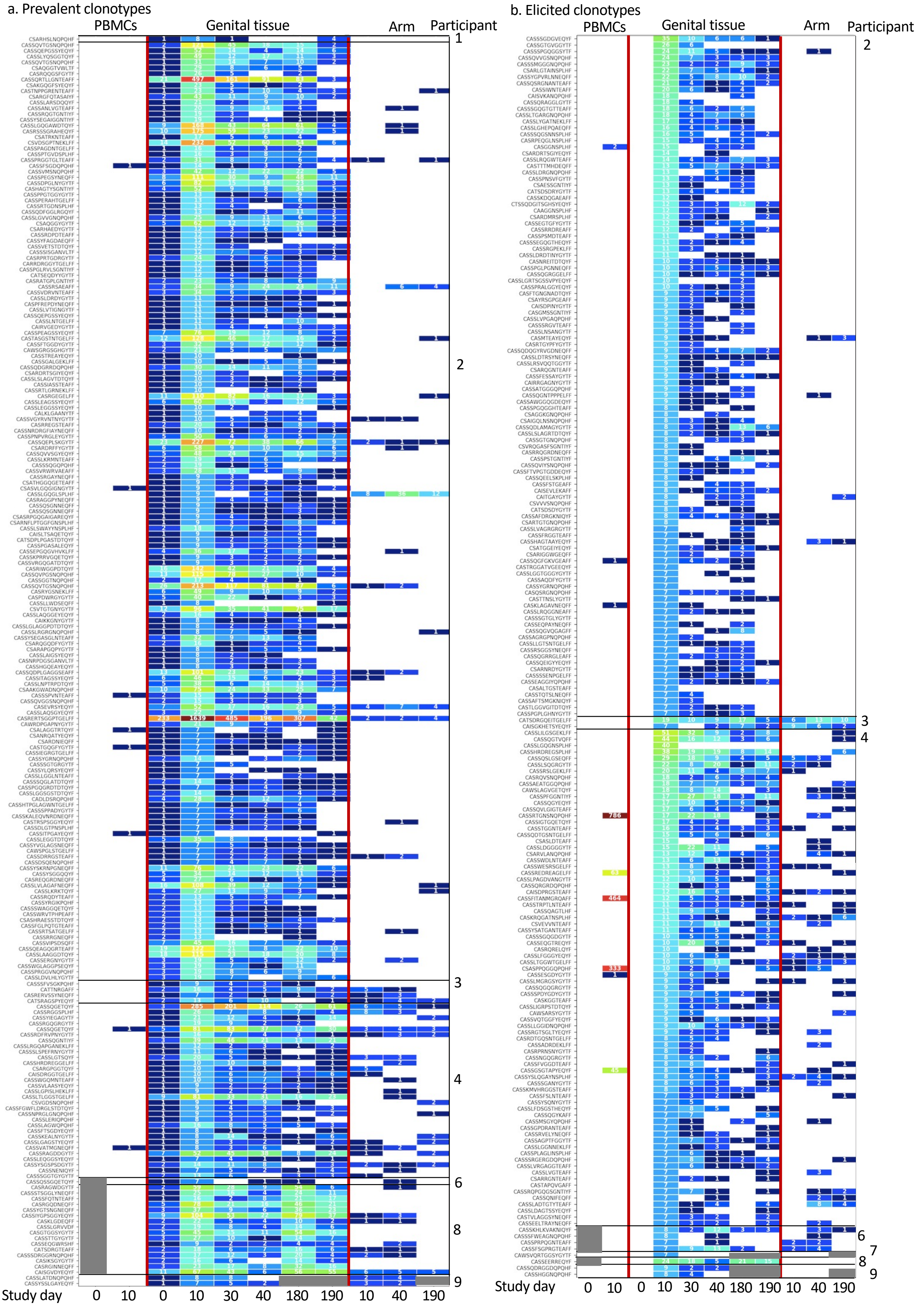

Supplementary Figure 2. All prevalent and elicited clonotypes expanding over dose 2

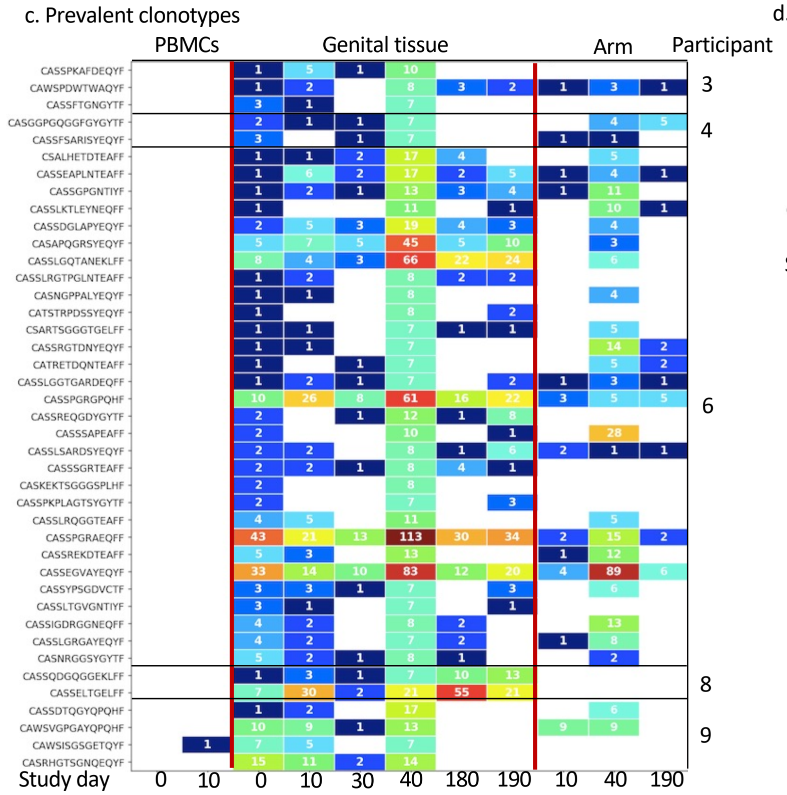

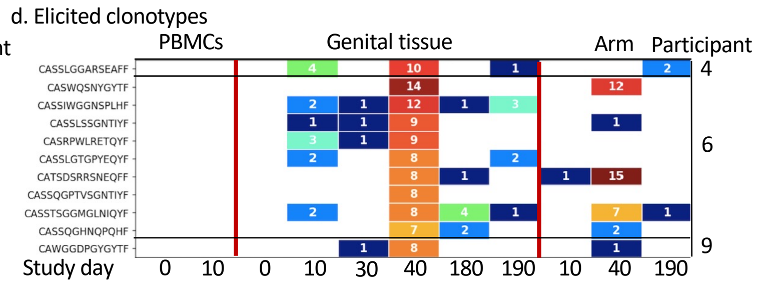

Supplementary Figure 3 – TRBV and TRBJ usage in all participants

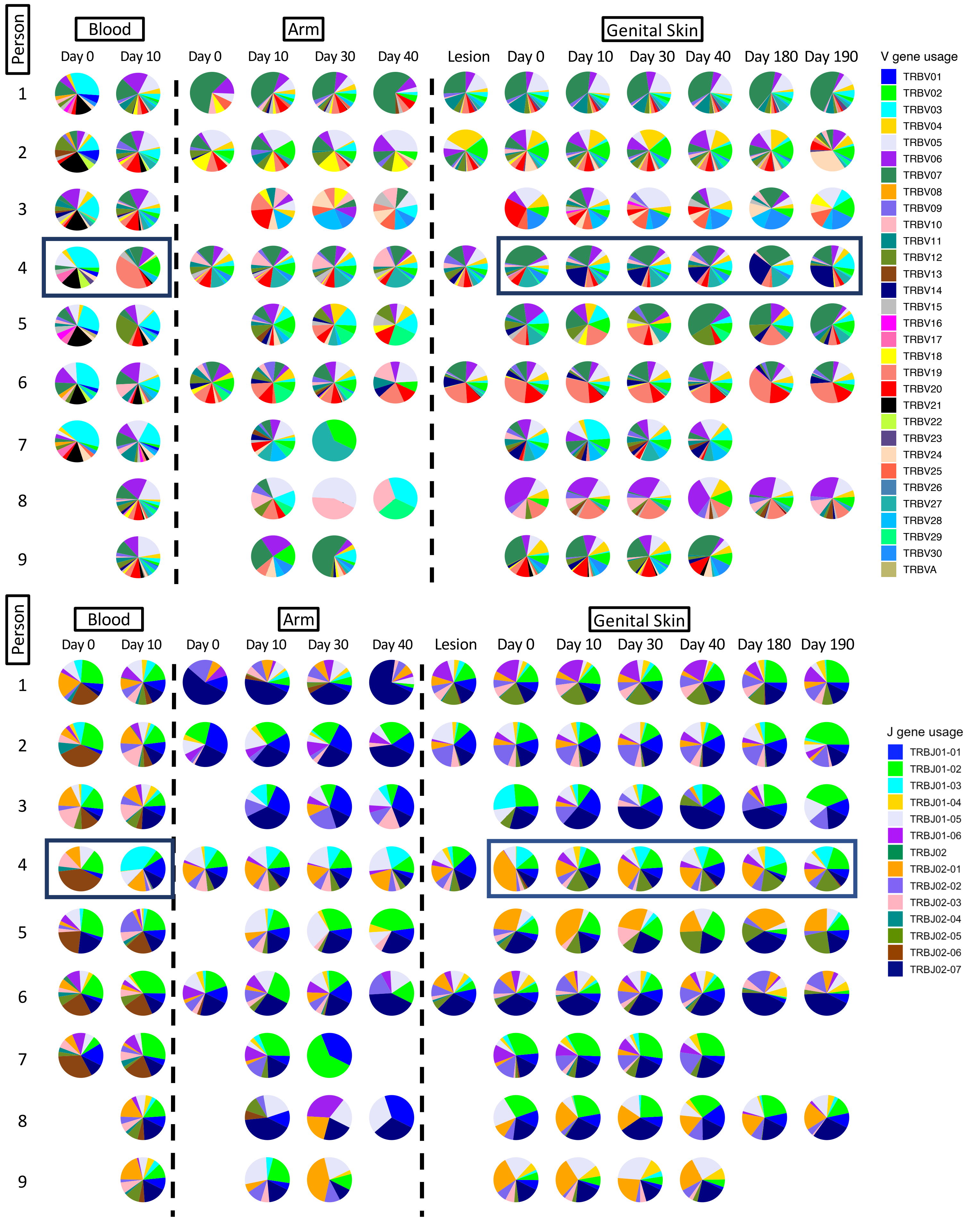

Supplementary Figure 4.

a.

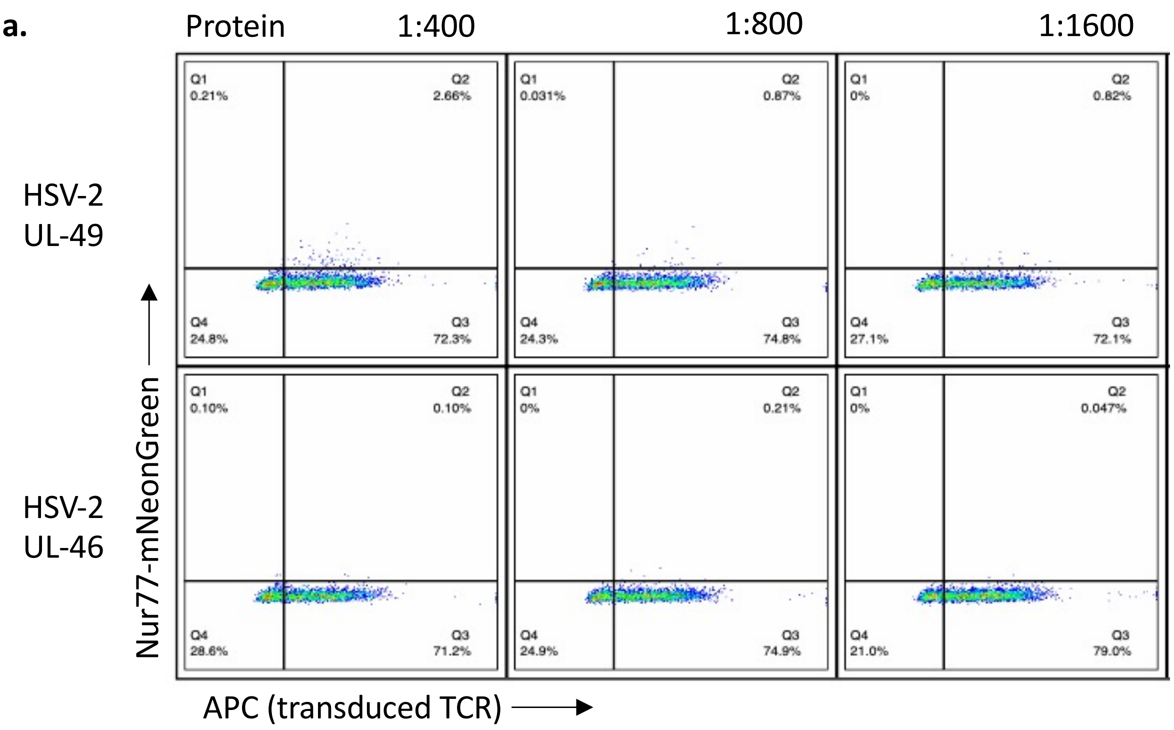

b.

81-93 ARPRRSASVAGSH

		Pool 9	Pool 10	Pool 11	Pool 12	Pool 13	Pool 14	Pool 15	Pool 16	Pool 17	Pool 18
	Pool 1	0.302	38.5	0.277	0.204	0.178	0.373	0.255	0.304	0.201	0.174
81-93	Pool 2	35.8	53.7	32.9	35.5	36.1	37.1	36.2	37.1	37.7	38.2
	Pool 3	8.21	41.7	5.25	5.56	5.95	5.97	7.75	6.62	6.89	7.82
	Pool 4	0.174	27.1	0.148	0.226	0.127	0.2	0.241	0.255	0.211	0.259
	Pool 5	0.358	32.3	0.25	0.257	0.126	0.101	0.16	0.233	0.0265	0.109
	Pool 6	0.232	40.8	0.154	0.129	0.104	0.183	0.0802	0.0524		0.27
	Pool 7	0.0518	39.7	0.154	0.257	0.176	0.283	0.321	0.208		
	Pool 8	0.129	32.3	0.103	0.256	0.0796	0.157	0.214			

c.

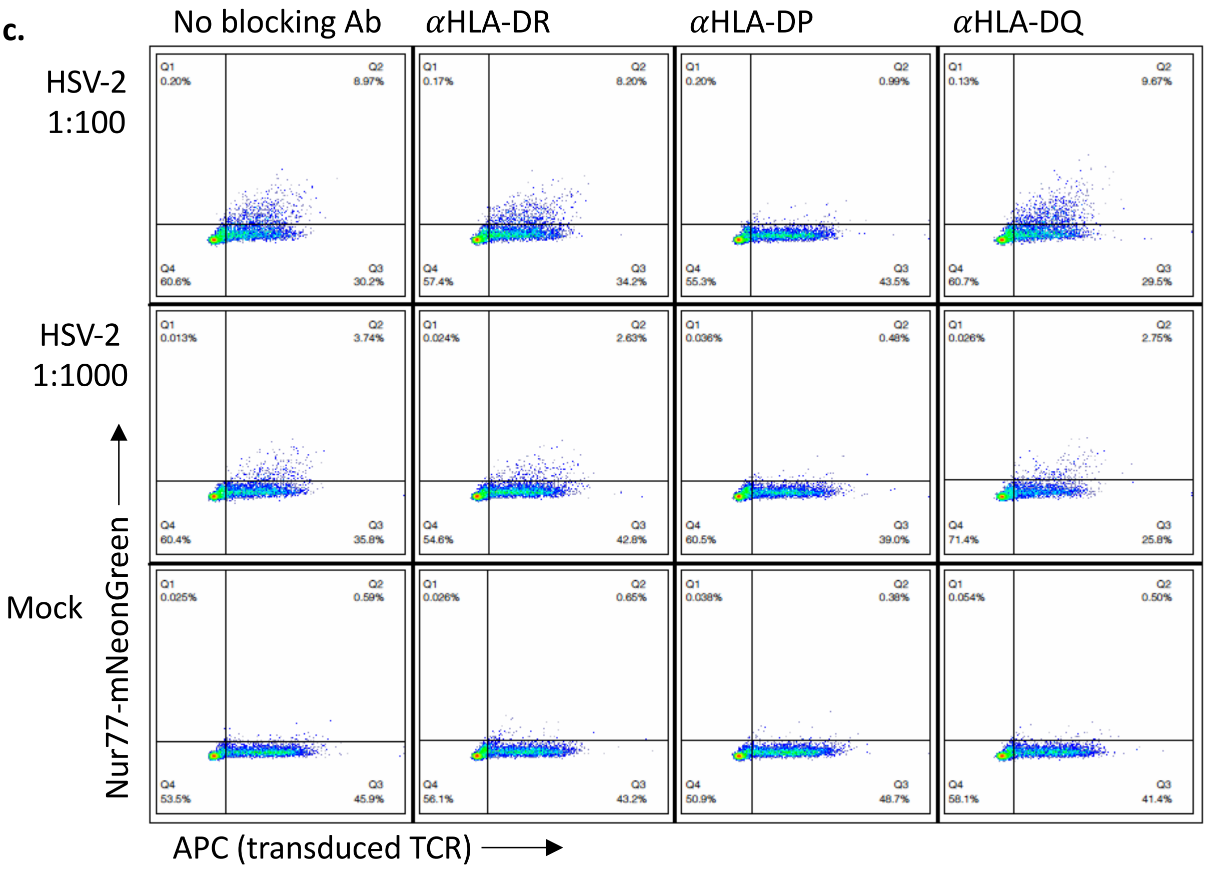

Supplementary Figure 5.

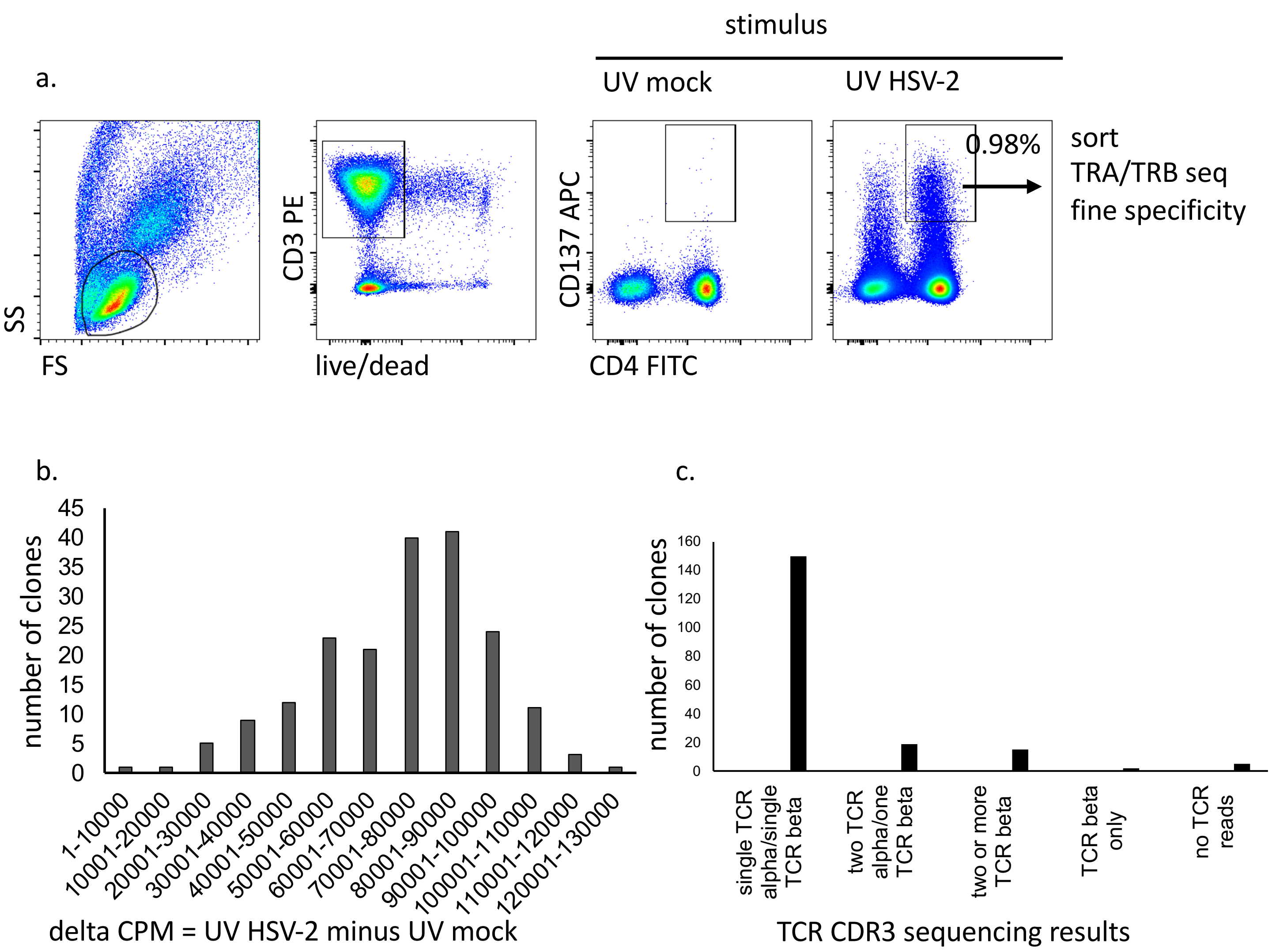

Supplement: Supplemental data [file jciinsight-9-179010-s157.pdf]
